# The Relationship between the Items of the Barthel Index and Short-Term Prognosis in Terminal Cancer Patients

**DOI:** 10.3390/reports6010005

**Published:** 2023-01-31

**Authors:** Shinya Okamoto, Kazuko Okazaki, Masahiro Okada, Fumiyoshi Murakami, Hiroki Sugihara, Yoshinori Hoshino, Yuka Ogawa, Kengo Banshoya, Eisuke Takei, Shuso Takeda, Narumi Sugihara

**Affiliations:** 1 Department of Pharmacy Onomichi Municipal Hospital, 3–1170–177 Shintakayama, Onomichi 722-8503, Japan; 2 Faculty of Pharmacy and Pharmaceutical Sciences, Fukuyama University, Sanzou 1, Hiroshima 729-0292, Japan

**Keywords:** Barthel Index, feeding, mobility, grooming, prognostic prediction, terminal cancer patients

## Abstract

Predicting the short-term prognosis of patients with terminal cancer is important for treatment decisions and improving patients’ quality of life. Recently, it has been reported that the Barthel Index (BI) can predict short-term prognosis. This study aimed to distinguish the BI items that can more accurately predict the short-term prognosis of terminal cancer patients from among the other BI items. This study compared the accuracy of predicting the 1-, 2-, and 3-week prognosis of BI and individual BI items in 158 cancer patients who died between January 2018 and June 2020 at the Onomichi Municipal Hospital in Japan. For predicting the 1- and 2-week prognosis, the BI item “feeding” scores of 0/5 and 10 showed higher accuracies (0.766 and 0.715, respectively) than BI scores between 0–15/20–100. For predicting a 3-week prognosis, the BI item “mobility” scores of 0, 5/10, 15 and the BI item “grooming” scores between 0/5 showed higher accuracies (0.627 and 0.614, respectively) than BI scores between 0–35/40–100. BI and individual BI items may be an option for prognostic prediction in terminal cancer patients.

## 1. Introduction

Terminal cancer patients and their families want prognostic information [1]. In addition, information about prognostic predictions for terminal cancer patients is helpful for medical staff to provide optimal treatment [2,3]. To estimate the short–term prognosis of patients with terminal cancer, it is important to develop prognostic indicators that do not require blood tests [4].

The Barthel Index (BI) is a commonly used Activities of Daily Living (ADL) index [5]. Godfrey et al. reported that determining the Barthel score on admission can predict a short prognosis in palliative care patients [6]. We reported that the BI might be more useful as a prognostic indicator than the Glasgow Prognostic Score in terminal cancer patients [7]. Further, we reported the relationship differences between individual components of the BI and the mortality of terminal cancer patients [8]. It was considered that some BI items were more suitable for short-term prognosis prediction, while others were unsuitable for short-term prognosis prediction in patients requiring palliative care.

To the best of our knowledge, this is the first study to explore BI and individual BI items as short–term prognosis indicators for patients with terminal cancer. Therefore, the aim of this study was to identify BI items that can more accurately predict the short-term prognosis of terminal cancer patients from among the BI items.

## 2. Materials and Methods

### 2.1. Patients

We retrospectively analyzed information on patients with cancer as their primary diagnosis who died while hospitalized at Onomichi Municipal Hospital in Japan from January 2018 to June 2020. Patients with cancer as their primary diagnosis were determined using the Diagnosis Procedure Combination claims database. The background of the patients was investigated with respect to age, sex, primary cancer, and duration between admission and death.

### 2.2. BI evaluation Methods

The BI is one of the most widely used ADL measures for independence. The BI consists of 10 items: feeding, mobility, grooming, toilet use, bathing, transfer, stairs, dressing, bowels, and bladder, which uses an ordinal rating scale (0, 5, 10, or 15) to give a total possible score of 0 to 100 [5]. To investigate the predictive accuracy of the BI, we divided the BI into five groups by score: “BI 0–15,” “BI 20–35,” “BI 40–55,” “BI 60–75,” and “BI 80–100” [9].

### 2.3. Predictive Performance

**Figure 1:**
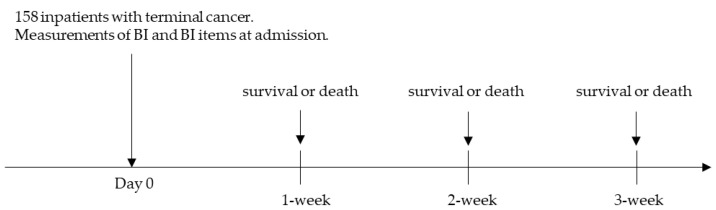
The procedure for evaluating the prognostic accuracy of BI in patients with terminal cancer. (Abbreviations: BI, Barthel index.)

This study aimed to investigate the predictive accuracy of BI and individual BI items at admission. Thus, the sensitivity, specificity, positive predictive value (PPV), negative predictive value (NPV), and accuracy for predicting 1-, 2- and 3-week prognosis of BI and BI items at hospitalization were calculated (Figure 1). Accuracy was calculated by dividing the sum of the true positive and true negative cases by the total number of cases.

### 2.4. Statistical Analysis

Cutoff values for prognosis prediction were set by the plot of a receiver operating characteristic curve with the highest Youden index for BI and individual BI items. Survival or death within 1, 2, and 3 weeks of admission was defined as dependent items, and BI and BI items were defined as independent items.

Statistical analyses were performed using EZR version 1.40 (Saitama Medical Center, Jichi Medical University, Saitama, Japan) [10].

With reference to Baba et al., we calculated the required sample size. The sample size was assumed to be a minimum 150 patients, with all study results required to calculate an accuracy within 15% width at 95% confidence intervals for a value of 70% [11].

## 3. Results

A total of 165 patients were enrolled, and 158 were selected after excluding 7 with missing BI data. Characteristics are shown in Table 1. The median (25–75% interquartile range (IQR)) age was 79 (71–85) years. The proportions of men and women were 66.5% and 33.5%, respectively. The median (IQR) duration from admission to death was 20 days (range: 10–37 days).

Table 2 shows the sensitivity, specificity, PPV, NPV, and accuracy of the BI and individual BI items. For predicting 1-week prognosis, using a range of BI scores between 0–15/20–100 demonstrated accuracy (0.633). For predicting 2-week prognosis, using a range of BI scores between 0–15/20–100 demonstrated accuracy (0.633). For predicting 3-week prognosis, using a range of BI scores between 0–35/40–100 demonstrated accuracy (0.608). For predicting 1- and 2-week prognosis, the BI item “feeding” score between 0/5, 10 showed higher accuracy (0.766 and 0.715, respectively) than the range of BI scores between 0–15/20–100. Furthermore, for predicting 3-week prognosis, the BI item “mobility” scores between 0, 5/10, 15 and the BI item “grooming” scores between 0/5 showed higher accuracy (0.627 and 0.614, respectively) than BI scores between 0–35/40–100.

## 4. Discussion

Godfrey et al. and Bennett et al. and our previous studies have shown that decreased BI score is an important prognostic indicator [6,7,8,12]. From these reports, we hypothesized that a decrease in a specific BI item score may have an effect on the BI score decrease.

This study revealed the characteristics of individual BI items as short–term prognostic indicators for terminal cancer patients. The results of this study suggest that the BI item “feeding” can better predict 1- or 2-week prognosis for terminal cancer patients than BI. Moreover, it is suggested that the BI item “mobility” and “grooming” can be better predictors of 3-week prognosis in terminally ill cancer patients than BI.

In this study, the BI was scored by nurses. Because this was a retrospective study, the time required for BI scoring could not be ascertained. Anyone can easily evaluate BI by learning the measurement method from a manual (https://www.sralab.org/sites/default/files/2017-07/barthel.pdf, accessed on 29 December 2022.). It has been reported that the BI can be scored in an average of 2.2 minutes per patient and is very easy to use [13,14]. Scoring only some items of the BI is even easier than scoring the BI. In these respects as well, prognosis prediction using some items of BI is considered to be more useful.

Chow et al. reported that routine blood tests are not recommended in terminal cancer patients [4]. Performance status (PS), oral intake, dyspnea, and delirium have been reported as prognostic factors that do not require blood testing [15,16]. Validated prognostic tools that do not require blood tests include Palliative Prognostic Index (PPI) [17,18,19,20,21], and Performance Status–Based Palliative Prognostic Index (PS-PPI) [22]. The PPI is calculated by PS based on the Palliative Performance Scale (PPS) [23], oral intake, edema, dyspnea at rest, and delirium. Also, the PS of the PS-PPI is based on the Eastern Cooperative Oncology Group PS [24]. The PPI and PS-PPI are calculated by summing the PS score and several symptom scores. From the above, prognosis prediction using only a few items of BI may be much easier than prognosis prediction using the PPI and PS-PPI.

This study has some limitations. First, it was a retrospective study. Second, our study was performed at a single facility. Thus, the results of our study cannot be generalized. Therefore, a prospective multicenter validation study is needed in the future. 

## 5. Conclusions

As a simple short-term prognostic method for terminal cancer patients that does not require a blood test, we reported on a survey that focused on BI items. The use of BI items such as “feeding”, “mobility” and “grooming” is worthwhile for predicting short-term prognosis in terminal cancer patients. 

## Figures and Tables

**Table 1:** Characteristics of the patients in this study.

Characteristic	Findings
N	158
Age (years)	79 (71–85)
Sex (men/women), n (%)	105/53 (66.5/33.5)
Primary cancer, n (%)	
Colorectal	25 (15.8)
Lung malignant mesothelioma	21 (13.3)
Gastric	21 (13.3)
Liver	17 (10.8)
Pancreatic	15 (9.5)
Biliary Tract	14 (8.9)
Blood	12 (7.6)
Brain	8 (5.1)
Prostate	5 (3.2)
Bladder	5 (3.2)
Others	15 (9.5)
Duration between admission and death (days)	20 (10–37)

Data are median (25–75% interquartile range) or percentage.

**Table 2:** Sensitivity, specificity, PPV, NPV, accuracy of BI and BI items.

	Item	Cutoff Value	Sensitivity	Specificity	PPV	NPV	Accuracy
1-week	BI	0–15/20–100	0.667	0.627	0.242	0.913	0.633
	Feeding	0/5,10	0.625	0.791	0.349	0.922	0.766
	Mobility	0,5/10,15	0.833	0.507	0.233	0.944	0.557
	Grooming	0/5	0.875	0.276	0.178	0.925	0.367
	Toilet use	0/5,10	0.750	0.575	0.240	0.928	0.601
	Bathing	0/5	0.958	0.179	0.173	0.960	0.297
	Transfer	0/5,10,15	0.833	0.448	0.213	0.938	0.506
	Stairs	0/5,10	0.792	0.321	0.173	0.896	0.392
	Dressing	0/5,10	0.792	0.463	0.209	0.925	0.513
	Bowels	0/5,10	0.667	0.567	0.216	0.905	0.582
	Bladder	0/5,10	0.667	0.575	0.219	0.906	0.589
2-week	BI	0–15/20–100	0.571	0.667	0.485	0.739	0.633
	Feeding	0/5,10	0.482	0.843	0.628	0.748	0.715
	Mobility	0,5/10,15	0.732	0.559	0.477	0.792	0.620
	Grooming	0/5	0.821	0.294	0.390	0.750	0.481
	Toilet use	0/5,10	0.607	0.598	0.453	0.735	0.601
	Bathing	0/5	0.893	0.186	0.376	0.760	0.437
	Transfer	0/5,10,15	0.714	0.471	0.426	0.750	0.557
	Stairs	0/5,10	0.821	0.373	0.418	0.792	0.532
	Dressing	0/5,10	0.661	0.471	0.407	0.716	0.538
	Bowels	0/5,10	0.589	0.598	0.446	0.726	0.595
	Bladder	0/5,10	0.571	0.598	0.438	0.718	0.589
3-week	BI	0–35/40–100	0.694	0.507	0.621	0.587	0.608
	Feeding	0,5/10	0.647	0.521	0.611	0.559	0.589
	Mobility	0,5/10,15	0.659	0.589	0.651	0.597	0.627
	Grooming	0/5	0.835	0.356	0.602	0.650	0.614
	Toilet use	0,5/10	0.859	0.315	0.593	0.657	0.608
	Bathing	0/5	0.894	0.219	0.571	0.640	0.582
	Transfer	0/5,10,15	0.682	0.507	0.617	0.578	0.601
	Stairs	0/5,10	0.776	0.397	0.600	0.604	0.601
	Dressing	0/5,10	0.659	0.521	0.615	0.567	0.595
	Bowels	0,5/10	0.682	0.493	0.611	0.571	0.595
	Bladder	0,5/10	0.706	0.466	0.606	0.576	0.595

Abbreviations: BI, Barthel index; PPV, positive predictive value; NPV, negative predictive value. The optimal prognostic cutoff values for the BI and individual BI items were determined by plotting a receiver operating characteristic curve with the highest Youden index among all possible cutoff values.

## Data Availability

The data presented in this study are available upon request from the corresponding author (Masahiro Okada).
